# A Computational Procedure for Assessing the Dynamic Performance of Diffusion-Controlled Transdermal Delivery Devices

**DOI:** 10.3390/pharmaceutics3030485

**Published:** 2011-08-11

**Authors:** Laurent Simon

**Affiliations:** Otto H. York Department of Chemical, Biological and Pharmaceutical Engineering, New Jersey Institute of Technology, Newark NJ 07102, USA; E-Mail: laurent.simon@njit.edu; Tel.: +1-973-596-5263; Fax: +1-973-596-8436.

**Keywords:** time constant, diffusion, mathematical modeling

## Abstract

The dynamic performances of two different controlled-release systems were analyzed. In a reservoir-type drug-delivery patch, the transdermal flux is influenced by the properties of the membrane. A constant thermodynamic drug activity is preserved in the donor compartment. Monolithic matrices are among the most inexpensive systems used to direct drug delivery. In these structures, the active pharmaceutical ingredients are encapsulated within a polymeric material. Despite the popularity of these two devices, to tailor the properties of the polymer and additives to specific transient behaviors can be challenging and time-consuming. The heuristic approaches often considered to select the vehicle formulation provide limited insight into key permeation mechanisms making it difficult to predict the device performance. In this contribution, a method to calculate the flux response time in a system consisting of a reservoir and a polymeric membrane was proposed and confirmed. Nearly 8.60 h passed before the metoprolol delivery rate reached ninety-eight percent of its final value. An expression was derived for the time it took to transport the active pharmaceutical ingredient out of the polymer. Ninety-eight percent of alpha-tocopherol acetate was released in 461.4 h following application to the skin. The effective time constant can be computed to help develop optimum design strategies.

## 1. Introduction

Controlled-release devices are manufactured to deliver a specific dosage of a medication over an extended period of time. Some of the advantages of transdermal drug-delivery systems (TDDS) are improved patient compliance and a smooth plasma drug concentration profile as compared to oral administrations. In the development of TDDS, reservoir and monolithic (matrix) type devices are two main designs that have received increased attention. In the reservoir system, the drug is enclosed in a compartment located between a backing layer and a membrane that is used to control the delivery rate. The drug diffusion coefficient (D), and the thickness and compositions of the polymer can be manipulated to achieve a desired flux. Fick's second law of diffusion is implemented to describe drug transport across the rate limiting barrier. A lag effect is usually observed in these devices and equations are available in the literature to estimate physicochemical parameters necessary to simulate the process [1]. In the matrix system, the drug molecules are dissolved or dispersed throughout the membrane. Fabrication methods are designed to achieve a homogeneous distribution of the particles. Mathematical treatments of these products can be found in several modeling contributions [2-4].

The analysis conducted in this work takes the skin membrane into account. Drug transport is affected by resistances in the polymer and skin membranes. Because a constant thermodynamic activity is sustained in the donor, a steady-state permeation rate is achieved when reservoir devices are employed. Governing equations are derived that help explain the concentration profiles in terms of physicochemical parameters of the reservoir-polymer-skin system (hereafter called TDDS 1). Closed-form solutions can be obtained using techniques, such as the Residue method, to simulate the effects of membrane and drug properties on the release profile. These factors are expected to influence both the steady-state release rate (*j_ss_*) and the time it takes to attain the desired *j_ss_*. Although the concept of an effective time constant (*t_eff_*) has been applied to characterize single-layer heat conduction [5] and double-layer drug-release systems [6], an analytical expression has not been formulated in the literature for the TDDS 1. Such an equation would allow researchers and manufacturers of transdermal patches to select excipients needed to make sure that the target delivery rate is achieved within a prescribed time.

When the release kinetics is influenced by both, the stratum corneous and a monolithic film, a steady-state flux is not attained in the absence of a reservoir. Instead, the fraction of drug released from the matrix is monitored. The drug transport mechanism in these matrix-skin systems (hereafter called TDDS 2) has been studied by several researchers [2,4]. It is clear, from the findings, that factors, such as the polymer-skin partition coefficient and the drug diffusivities in the vehicle and the skin, direct the fractional release profiles. However, the time required for the drugs to be released from the matrix is usually not known *a priori*. This metric would help estimate when to replace the monolithic patch. Research shows that repeated applications of the device can be used as a strategy to maintain a steady-state flux [7], leading to an effective drug concentration in the blood stream [8,9]. Similar to the TDDS 1 device, a single time constant can also be derived to predict the time (*t_eff_*) it takes to release the drugs from the matrix.

This contribution focuses on the calculation of *t_eff_* for TDDS 1 and 2. The article is organized as follows: A brief description of the governing transport equations is provided. Section 2 describes published experimental data that are used to illustrate the applications *t_eff_* in predicting how fast an equilibrium flux is achieved in TDDS 1 or the time required to release 98% of the drug in TDDS 2. The results are presented and discussed in Section 3.

### 1.1. Transport equations in TDDS 1

The transport equations through both devices have been applied in several publications. These expressions are repeated here for completeness and to lay the foundation for the approach proposed in the Results and Discussion section. The temporal change in drug concentrations in the membrane, *C*_1_, and in the skin, *C*_2_ are:

(1) $$\frac{\partial C_{1}}{\partial t}=D_{1}\frac{\partial^{2}C_{1}}{\partial x^{2}},\;-l_{a}\leq x\leq 0$$

(2) $$\frac{\partial C_{2}}{\partial t}=D_{2}\frac{\partial^{2}C_{2}}{\partial x^{2}},\;0\leq x\leq l_{b}$$

where *D*_1_ and *D*_2_ are the diffusion coefficients in the polymeric and dermal membranes, respectively. The following initial conditions apply in the two layers:

(3) $$C_{1}\left(x,0\right)=0,\;-l_{a}\leq x\leq 0$$

and

(4) $$C_{2}\left(x,0\right)=0,\;0\leq x\leq l_{b}$$

The boundary conditions associated with Eqs. (1) and (2) take the forms:

$$C_{1}\left(-l_{a},t\right)=C_{0}$$

(6) $$D_{1}\frac{\partial C_{1}}{\partial x}\left(0,t\right)=D_{2}\frac{\partial C_{2}}{\partial x}\left(0,t\right)$$

(7) $$C_{1}\left(0,t\right)=k_{m}C_{2}\;\left(0,t\right)$$

and

(8) $$C_{2}\left(l_{b},t\right)=0$$

Equation (5) relates the concentration at −*l*_a_ to the donor concentration. The infinite source condition is adequate for applications in which the loading dose is well above the drug saturation limit in the donor. The equilibrium partition coefficient at *x* = −*l*_a_ is assumed to have a value of 1; Eq. (6) describes the continuity of flux at the membrane-skin boundary; Eq. (7) denotes an equilibrium condition at *x* = 0 where *k_m_* is the partition coefficient. A perfect sink condition is expressed by (8).

### 1.2. Transport equations in TDDS 2

The governing equations are similar to the ones describing drug transport through TDDS 1 except for the following changes. Equation (3) is replaced by:

(9) $$C_{1}\left(x,\;0\right)=C_{0},-l_{a}\leq x\leq 0$$

to express that the drug is uniformly distributed in the membrane. Equation (5) becomes a zero-flux condition:

(10) $$\frac{\partial C_{1}\left(-l_{a},t\right)}{\partial x}=0$$

indicating that no mass is exchanged with the environment.

### 1.3. The notion of an effective time constant

A first-order system can be written as:

(11) $$\tau_{p}\;\frac{dy}{dt}+y=K_{p}u\left(t\right)$$

where the model parameters *τ_p_* and *K_p_* are the time constant and steady-state gain of the process. The variables *u* and *y* denote the input and output (or response) variables. While *K_p_* represents the ratio of the equilibrium response (*y_ss_*) to the size of a step change in *u*, *τ_p_* is a measure of how long it takes to reach *y_ss_*. The gain *K_p_* is a measure of the sensitivity of a system. For example, consider a process where saturated steam is supplied to heat the liquid in a vessel. A *K_p_* value of 5.0 °F/(lb/min) implies that an increase of 1.0 lb/min in the steam mass flow rate is needed to raise the liquid temperature by 5.0 °F.

It can be shown that *y* is at 63.2% of its ultimate value after one time constant. At 4*τ_p_* , the response has attained 98% of its final value (called the response time). In the tank heater case, the response time denotes the period elapsed before the temperature changes by 5.0 °F. Using the Laplace variable *s*, often used to analyze the dynamics of linear systems, the response becomes:

(12) $$Y\left(s\right)=\frac{K_{p}}{\tau s+1}U\left(s\right)$$

where *Y* and *U* represent the Laplace transforms *u* and *y* assuming that *y*(0) = *u*(0) = 0. For example, a unit step change in *u* leads to:

(13) $$Y\left(s\right)=\frac{K_{p}}{s\left(\tau s+1\right)}$$

Without solving for *y*(*t*) directly in Eq. (11), an inspection of Eq. (12) shows the time constant. In addition, the steady-state value *y_ss_* is easily obtained:

(14) $$y_{ss}=\underset{s\to 0}{\lim}sY\left(s\right)=\underset{s\to 0}{\lim}s\frac{K_{p}}{s\left(\tau s+1\right)}=K_{p}$$

after applying the final value theorem.

A similar approach can be adopted for processes in which the variable of interest can be approximated by a series:

(15) $$\psi \left(x,t\right)=\sum_{n=1}^{\infty }f_{n}\left(x\right)\exp\left(-\lambda_{n}t\right)$$

where *λ_n_* = 1/*t_n_* and *f_n_* is a function of *x*. The numbers *t_n_* designate characteristic time constants (*t_n_* > *t_m_* or *λ_n_* < *λ_m_* for *n* < *m*). In general, the system dynamics is represented by the first *λ_n_* values. To employ a single time constant that estimates how fast *ψ(x,t)* approaches the equilibrium *ψ_ss_*, a first-moment relaxation time constant is applied:

(16) $$t_{eff}=\underset{s\to 0}{\lim}\left(\frac{\psi_{ss}\left(x\right)}{s^{2}}+\frac{d\bar{\psi }\left(x,s\right)}{ds}\right)\;{\left[\underset{s\to 0}{\lim}\left(\frac{\psi_{ss}\left(x\right)}{s}-\bar{\psi }\left(x,s\right)\right)\right]}^{-1}$$

where *t_eff_* is a function of position *x*. Note that, for this investigation, *t_eff_* is independent of *x* because *ψ* is defined as the delivery rate or the fraction of drug released. The derivation of *t_eff_* and other applications of this approach are found in [5]. Equation 15 is obtained by solving the two-layer diffusion problem using Laplace transform methods or the technique of separation of variables. When the former procedure is implemented, Laplace transform expressions are readily available and can be used to derive steady-state conditions (*i.e.*, final value theorem). The representation of the solution, in terms of a single-infinite series, is based on the assumption of a one-dimensional solute transport. If a rectangular geometry was considered, a double-infinite series would be obtained.

## 2. Experimental Section

The experimental data used to analyze the TDDS 1 device are based on the release of metoprolol in hairless rats published in [10]. Drug transport was described in [11] and [12]. Metoprolol is known to help treat patients who suffer from hypertension and angina. Ouriemchi and Vergnaud examined a situation in which a constant concentration of 0.036 g/cm^3^ was maintained on the external side of a polymeric membrane 0.12-cm thick (Scotch Pack 1006, 3M Company, USA) [11]. The thickness of the skin was 0.038 cm and the drug diffusivity coefficients in the membrane and dermal layers were 4.4 × 10^−5^ cm^2^/s and 2.2 × 10^−8^ cm^2^/s, respectively. These parameters, when incorporated in a mathematical model, were able to simulate the permeation study conducted by Ghosh *et al.* [10].

Released data from the permeation of alpha-tocopherol acetate (ATA) were used to examine the performance of TDDS 2 [13]. The ester prodrug (ATA) is preferred over alpha-tocopherol (AT), a vitamin E homologue, for topical applications because of AT's particularly short shelf life. AT provides protection against mutagens and the deleterious effects of ultraviolet B (UVB) rays on the skin. Studies, conducted by Saral *et al.*, led to similar conclusions concerning the role of ATA in protecting dorsal skin of guinea pigs from UVB-induced damage [14]. Mahamonghol *et al.* focused on five formulations of ATA [13]. This contribution uses the results from the permeation of 5% (w/w) ATA from isopropyl myristate. The experiments occurred in a redesigned Franz diffusion cell system (Crown Glass Company, Somerville, NJ) maintained at 37 °C. Cadaver skins were placed between the donor and receiver chambers. The permeability of ATA through the skin was 1.1 × 10^−2^ cm/h; the thickness of the donor solution and the membrane were 0.56 cm and 0.005 cm, respectively. The initial concentration in the donor compartment was 50000 μg/cm^3^.

## 3. Results and Discussion

### 3.1. Derivation of t_eff_ for TDDS 1

The variables and original model equations are converted into their dimensionless counterparts:

(17) $$\begin{array}{c} U_{1}=\frac{C_{1}}{C_{0}}\;U_{2}=\frac{C_{2}}{C_{0}}\;\beta =\frac{D_{2}l_{a}}{D_{1}l_{b}}\;p=\frac{D_{1}l_{b}^{2}}{D_{2}l_{a}^{2}}\\ \tau =\frac{D_{2}}{l_{b}^{2}}t\;\chi_{1}=\frac{x}{l_{a}},\;-l_{a}\leq x\leq 0\;\chi_{2}=\frac{x}{l_{b}},\;0\leq x\leq l_{b} \end{array}$$

(18) $$\frac{\partial U_{1}}{\partial \tau }=p\frac{\partial^{2}U_{1}}{\partial \chi_{1}^{2}},\;-1\leq \chi_{1}\leq 0$$

(19) $$\frac{\partial U_{2}}{\partial \tau }=\frac{\partial^{2}U_{2}}{\partial \chi_{2}^{2}},\;0\leq \chi_{2}\leq 1$$

with the initial conditions given by:

(20) $$U_{1}\;\left(\chi_{1},0\right)=0$$

and

(21) $$U_{2}\;\left(\chi_{2},0\right)=0$$

Similarly, Eqs. (5) to (8) become:

(22) $$U_{1}\left(-1,\tau \right)=1$$

(23) $$\frac{\partial U_{1}}{\partial \chi_{1}}\left(0,\tau \right)=\beta \frac{\partial U_{2}}{\partial \chi_{2}}\left(0,\tau \right)$$

(24) $$U_{1}\left(0,\tau \right)=k_{m}U_{2}\;\left(0,\tau \right)$$

and

(25) $$U_{2}\;\left(1,\tau \right)=0\left(25\right)$$

If we define the normalized flux by:

(26) $$J\left(\tau \right)=-\frac{\partial U_{2}\;\left(1,\tau \right)}{\partial \chi_{2}}$$

The dimensional expression is:

(27) $$J_{t}=\frac{C_{0}D_{2}}{l_{b}}J\left(\tau \right)$$

It can be shown that the ratio of the flux to its steady-state value in the Laplace domain is:

(28) $$\frac{j\left(s\right)}{j_{ss}}=\frac{J\left(s\right)}{J_{ss}}=\frac{\beta +k_{m}}{\sqrt{s}\left[k_{m}\sinh\left(\sqrt{s}\right)\cosh\left(\sqrt{\frac{s}{p}}\right)+\beta \sqrt{p}\cosh\left(\sqrt{s}\right)\sinh\left(\sqrt{\frac{s}{p}}\right)\right]}$$

and after applying Eq. (16), the normalized and dimensional effective time constants are:

(29) $$\tau_{eff}=\frac{14\beta \left(p+3\right)\left(3p+1\right)k_{m}+\left(p\left(7p+30\right)+75\right)k_{m}^{2}+\beta^{2}\left(15p\left(5p+2\right)+7\right)}{60p\left(\beta +k_{m}\right)\left(\beta +\left(p+3\right)k_{m}+3\beta p\right)}$$

and

(30) $$t=\frac{l_{b}^{2}}{D_{2}}\tau_{\text{eff}}$$

### 3.2. Application of t_eff_ for TDDS 1 to experimental data

**Figure 1.**
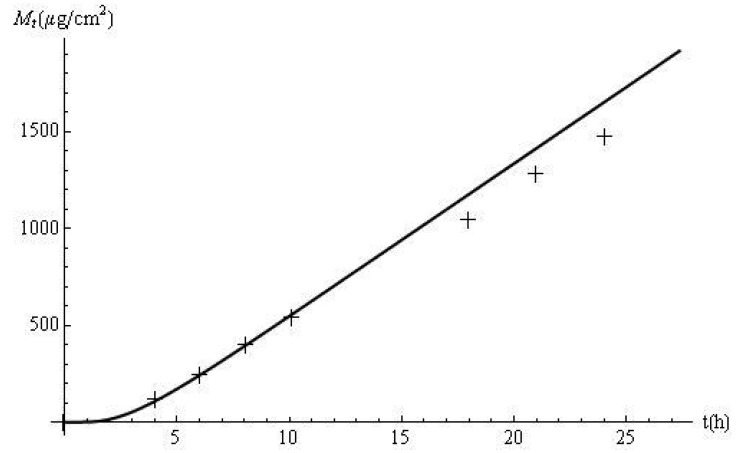
Cumulative amount of metoprolol released (experimental: +; predicted: −).

The model parameters were obtained from [11]: *C*_0_ = 0.038 g/cm^3^, *D*_1_ = 4.4 × 10^−5^ cm^2^/s, *D_2_* = 2.2 × 10^−8^ cm^2^/s, *l_a_* = 0.12 cm, *l_b_* = 0.038 cm and *k_m_* = 1 (*i.e.*, *β* = 1.58 × 10^−3^, *p* = 200.5). Data published in [10] were used to validate the mathematical approach. The experimental and calculated cumulative amounts of drug released:

(31) $$M_{t}=\int_{0}^{t}\left(J_{t}\right)dt$$

are depicted in Figure 1 to show that the theoretical model was able to predict the observations. An inversion method, developed in [15], was applied to obtain *J_t_* from Eq. (28). The discrepancy observed between the experimental and predicted data after 15 h may be due to parameter estimation errors.

**Figure 2.**
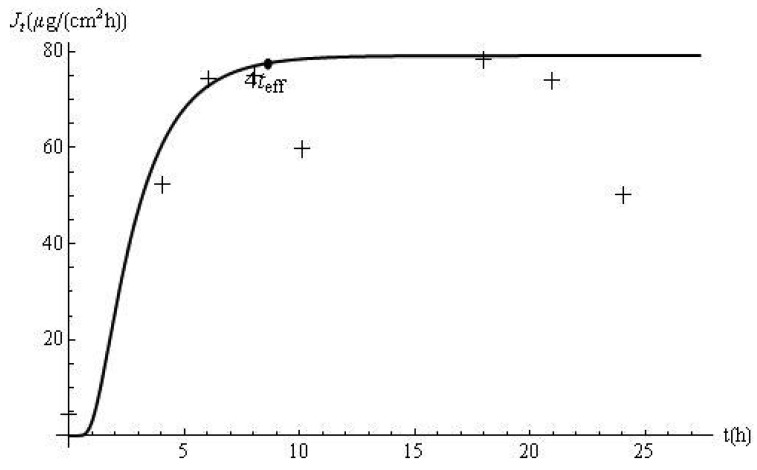
Metoprolol flux *versus* time (experimental: +; predicted: −).

The flux profiles are shown in Figure 2. The experimental delivery rate was obtained after applying a cubic spline to the data. The disagreement between the model predictions and the laboratory data, observed in Figure 1, is accentuated by the use of the derivative approximation. In this case, *t_eff_* and the response time (*i.e.*, 4*t_eff_*) are 2.15 h and 8.60 h, respectively. Based on this approach, it can be estimated, from the membrane and skin properties alone, that it should take approximately 8.60 h for the flux to reach 98% of its steady-state value of 79.2 μg/cm^2^ h. This prediction is confirmed by Figure 2. In addition, computations with *J_t_* show that the delivery at this time (*i.e.*, *J*_8.60_) is 77.62 μg/cm^2^ h, which is 98% of the ultimate value of the flux.

The time lag (*t_lag_*) is often calculated to determine the diffusion and partition coefficients for molecular transport through a flat membrane assuming perfect sink and infinite source conditions. In such a case, the steady-state flux is achieved after 2.7 × *t_lag_*. However, for the TDDS 1, the expression for *t_lag_* would involve the diffusion coefficients in the two layers, among other parameters. In addition, how this measurement is correlated with the onset of *j_ss_* is not as well established as in the case of the one-layer model. The effective time constant is adopted in this contribution.

### 3.3. Derivation of t_eff_ for TDDS 2

The normalized equations for TDDS 1 are also appropriate for TDDS 2 with the following modifications. Equations (20) and (22) are changed to:

(32) $$U_{1}\;\left(\chi_{1},0\right)=1\left(32\right)$$

(33) $$\frac{\partial U_{1}}{\partial \chi_{1}}\;\left(-1,\tau \right)=0$$

The cumulative amount of drug released is:

(34) $$M_{t}=-Al_{b}C_{1,0}\int_{0}^{\tau }\left(\frac{\partial U_{2}\left(1,\tau \right)}{\partial \chi_{2}}\right)d\tau$$

where *A* is the area exposed to the dosing solution. As a result, the Laplace transform of *M_t_* is [4]:

(35) $$M\left(s\right)=Al_{b}C_{1,0}\text{L}\frac{1}{s^{3/2}\left[k_{m}\sinh\left(\sqrt{s}\right)+\beta \sqrt{p}\cosh\left(\sqrt{s}\right)\coth\left(\frac{\sqrt{s}}{\sqrt{p}}\right)\right]}$$

Considering that:

(36) $$M_{\infty }=\underset{t\to \infty }{\lim}M_{t}\;\left(t\right)=s\underset{s\to 0}{\lim}M\;\left(s\right)=\frac{Al_{b}C_{1,0}}{p\beta }=Al_{a}C_{1,0}$$

the percentage of drug released is written as:

(37) $$\frac{M\left(s\right)}{M_{\infty }}=\frac{\beta p}{s^{3/2}\left[k_{m}\sinh\left(\sqrt{s}\right)+\beta \sqrt{p}\cosh\left(\sqrt{s}\right)\coth\left(\frac{\sqrt{s}}{\sqrt{p}}\right)\right]}$$

and Eq. (16) yields:

(38) $$\tau_{\text{eff}}=\frac{20\beta \left(5p+4\right)k_{m}+120k_{m}^{2}+\beta^{2}\left(5p\left(5p+4\right)+16\right)}{20\beta p\left(6k_{m}+\beta \left(3p+2\right)\right)}$$

with

(39) $$t_{eff}=\frac{l_{b}^{2}}{D_{2}}\tau_{eff}$$

### 3.4. Application of t_eff_ for TDDS 2 to experimental data

**Figure 3.**
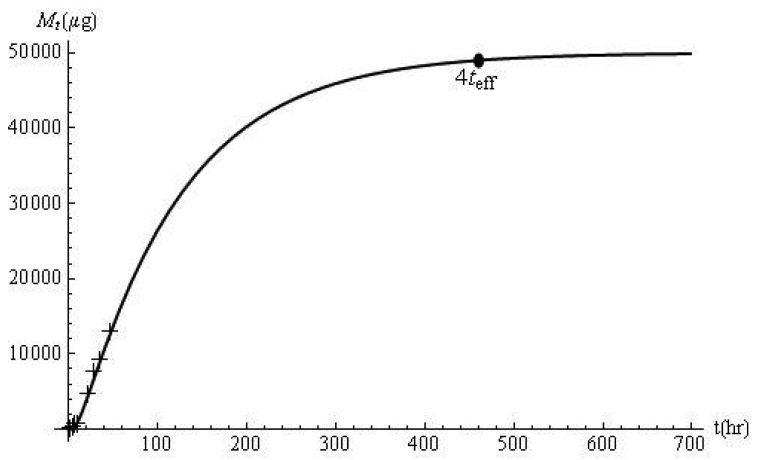
Cumulative amount of ATA released (experimental: +; predicted: −) and the response time.

Mahamongkol *et al.* (2005) provided permeation data which were analyzed, via the method outlined in [16], to determine the following parameters: *D*_1_ = 2.27 × 10^−3^ cm^2^/h, *D_2_* = 4.30 × 10^−7^ cm^2^/h, *k_m_* = 7.81 × 10^−3^ Additional data were obtained from [13]: *l_a_* = 0.56 cm, *l_b_* = 0.005 cm and *C*_0_ = 50000 μg/cm^3^ (*i.e.*, *β* = 3.81, *p* = 0.41). Good agreement was observed between predicted and experimental *M_t_* profiles measured in the first 50 min (Figure 3). Based on a calculated *t_eff_* of 115.3 h, it would take 461.4 h to release 98% of ATA from the device (*i.e.* 0.98 A*l_a_C_1,0_* = 49,000 μg). The same fraction of drug was estimated by calculating *M_t_*/*M_∞_* at *t* = 461.4 h.

Product designers can use the effective time constant to estimate the performances of TDDS 1 and TDDS 2. The method provides manufacturers with an analytical tool to help estimate the time it takes the medication to begin working after the application of a patch of type TDDS 1. In studies involving removal and reapplication of transdermal matrix systems [8,9,17], the method developed for TDDS 2 can be adapted to estimate how long a person has to wear the patch. In addition, calculation of *t_eff_* is straightforward and does not involve the solution of a system of partial differential equations. This approach can be applied to help design novel drug-delivery products where both the steady-state delivery rate and the effective time constant are controlled. The methods implemented do not rely on a short-time approximation of the original equations. Instead, the complete transient behavior of the process is included in the analysis. As a result, the effective-time-constant technique offers broad practical applications and may help researchers assess both short-term and long-term dynamic performances of a drug-delivery device. The framework also provides the possibility for investigating the effects of design specifications on *t_eff_*. Diffusion coefficients and the membrane thickness can be manipulated to produce a desired *t_eff_* value and satisfy end-user requirements. Because closed-form expressions are provided, sensitivity analysis can be conducted to identify which parameters influence the release time of a drug the most.

## 4. Conclusions

The time to reach a steady-state flux and to release the drug from the polymer matrix was derived for two types of devices TDDS 1 and TDDS 2. Although the methodology used the original partial differential equations (PDEs) and Laplace transforms, the final formulas contain user-friendly expressions that can be easily coded in spreadsheet packages. The effective time constant computed for the reservoir-polymer-skin system (TDDS 1) correctly predicted the period required to attain an equilibrium steady-state delivery rate of metoprolol in hairless rats. Analysis of data collected from the delivery of alpha-tocopherol acetate in an isopropyl myristate vehicle shows that it would take 461.4 h to release 98% of the drug. This estimation was in agreement with the numerical solution of the governing PDEs. The approach can be implemented in cases such as repeated applications of a patch and the optimal design of controlled release devices.
